# Potential association between allergenic food exposure and skin disease in Bangladesh: An application of principal component logistic regression (PCLR)

**DOI:** 10.1002/hsr2.70110

**Published:** 2024-09-29

**Authors:** Md. Abrar Ashfaq Khan, Md. Rashed Babu, Sumaiya Tasnim, Atiya Tarannum, Mohammad Anamul Haque, Nahid Sultana, Mohammad Ohid Ullah

**Affiliations:** ^1^ Department of Statistics Shahjalal University of Science and Technology Sylhet Bangladesh

**Keywords:** allergenic foods, Bangladesh, beef, brinjal, corn, hilsa, principal component logistic regression (PCLR), skin disease

## Abstract

**Background and Aim:**

Allergenic foods can trigger skin conditions, yet their impact in Bangladesh remains underexplored. This study aims to investigate the potential association between exposure to allergenic foods and the development of skin disease across Bangladesh.

**Methods:**

We conducted a cross‐sectional survey with 970 respondents from the divisions of Dhaka, Sylhet, Rangpur, and Chattogram who self‐reported skin disease triggered by food allergens. We analyzed the data using multiple response analysis and principal component logistic regression.

**Results:**

Approximately 64% of respondents had skin disease attributable to allergenic foods, with a relatively higher proportion among females (52%) than males (48%). Beef (67%), Brinjal (47.1%), Hilsa (45.8%), and Shrimp (23.7%) were the most frequently cited allergenic foods. Principal component logistic regression identified two highly significant principal components: the first representing Hilsa, Beef, and Brinjal (odds ratio = 2.256), and the sixth reflecting Corn (odds ratio = 1.342). Individuals who consumed foods with high loadings of the first principal component were 125.6% more likely to develop skin disease than nonconsumers, while those exposed to Corn had a 34.2% higher risk.

**Conclusion:**

The findings highlight prevalent allergenic foods in Bangladesh and their substantial impact on skin health, underscoring the need for dietary awareness and tailored interventions to mitigate allergic skin conditions in this population.

## 1 INTRODUCTION

Food is essential for survival, yet certain types of food may induce allergies or even result in life‐threatening reactions.^1^ While non‐immunologic factors produce food intolerance, it is an aberrant immunologic reaction to antigens delivered through food that causes food allergy (FA).^2^ Consequently, these allergenic foods increase the risk of several organ‐system‐specific allergic diseases.^3^ Symptoms of FA can range from minor skin issues to severe systemic responses. Particularly in conditions such as atopic dermatitis, allergens can penetrate damaged skin barriers—a process known as epicutaneous sensitization. This pathway helps FAs grow by initiating immune responses through skin‐derived dendritic cells and type 2 innate lymphoid cells. Therefore, this shows a very important link between skin diseases and FAs.^4^

Given this connection, it is not surprising that eating allergenic foods is a significant factor in promoting skin disease.^5^ Hence, it is crucial to recognize the specific foods through which FAs affect various demographics and contribute to other health issues, such as skin disease. In Asian countries like India and Bangladesh, allergies to spices such as grains, legumes, vegetables, red chili, ginger, coriander, cumin, mustard seed, and curry components like beef and brinjal are frequently noted.^6^ This variability in allergenic foods across regions is further illustrated by the differing prevalence rates of egg and peanut allergies in South Africa, which are 1.9% and 0.8%, respectively, while cow's milk allergy is less prevalent, predicted at only 0.1%.^7^ While dietary habits influence regional FA patterns, it is important to note that vulnerability to these allergies can also vary widely among different age groups and health conditions. The introduction of diverse foods in early childhood is a crucial period for the development or prevention of FAs.^8^ In fact, due to their immature immune systems and the manner and timing of food introduction, along with the body's developmental stage, children are more likely than adults to develop allergies prompted by specific foods.^8^, ^9^

Building upon the understanding of how allergenic foods can influence health across various demographics, research on how allergenic foods prompt skin disease in Bangladesh has unveiled significant findings. About 55% of the respondents in Tangail, Bangladesh, were reported to have a history of allergies in their families, with brinjal identified as the most common allergen, affecting 28.3% of the population studied.^10^ Furthermore, a study based on an online survey among university students from Bangladesh provided additional insights into dietary habits, revealing that higher dietary diversity is associated with higher socioeconomic status, older age, and fitness goals.^11^

Despite the global recognition of allergenic foods and their impact on skin disease, it is worth noting that research in Bangladesh specifically investigating these relationships has been notably limited. Previous studies focused on single locations, which may not accurately reflect national conditions. Additionally, to the best of our knowledge, before this study, hardly research had systematically examined the effects of allergenic foods on the skin health of the Bangladeshi population using a comprehensive approach that incorporates analyses like multiple response analysis, principal component analysis (PCA), and principal component logistic regression (PCLR). This gap underscores the critical need for a more detailed analysis that can address the diverse responses to food allergens across different demographics and geographic locations within the country. As a result, the primary objective of our research is to ascertain allergenic food exposures and their potential association with skin disease using exploratory data analysis and PCLR.

## 2 METHODOLOGY

### 2.1 Sampling technique

To collect data for this study, we conducted a cross‐sectional survey throughout Bangladesh using a purposive quota sampling technique. We chose this method because it effectively selects respondents from sub‐groups who can provide the most useful and relevant information about the research subject.^12^ In implementing this approach, the sub‐groups under the quota were based on geographic regions, including the divisions of Dhaka, Sylhet, Rangpur, and Chattogram, selected as mutually exclusive categories to ensure a broad geographic representation across the country. Given the lack of existing administrative data on the prevalence of skin disease caused by allergenic foods, the sample sizes for each division were determined based on the primary investigator's judgment.

### 2.2 Variables

In designing our survey, we utilized two key dichotomous variables to determine the inclusion criteria for further investigation. First, we defined the variable “Presence of Skin Disease,” with categories “Yes = 1” and “No = 0,” and queried respondents about the existence of any skin disease during the survey. Second, we introduced the variable “Food Exposure,” similarly categorized as “Yes = 1” and “No = 0,” which inquired from respondents whether any specific food item triggered their reported skin disease. This dual‐variable approach ensured that only respondents who answered “Yes” to both questions were included for further detailed surveying. To systematically evaluate potential associations with skin disease outcomes among the respondents, we incorporated specific food items into the questionnaire as categorical exposure factors. These food items, identified as potential triggers of skin disease, namely Hilsa, Shrimp, Brinjal, Spinach, Arum, Lentil, Corn, Beef, Mutton, Duck, Pineapple, Chicken's egg, and Duck's egg, were derived from key findings in previous studies.^10^, ^13^, ^14^, ^15^, ^16^, ^17^, ^18^, ^19^, ^20^

### 2.3 Data collection

Notably, studies in developing countries often depend on self‐reported data to diagnose food allergies due to several restrictions, such as a lack of diagnostic equipment, specialized healthcare practitioners, financial resources, and other similar issues.^21^ Recognizing these challenges, our approach aimed to capture a balanced and representative sample from each division, targeting approximately 200 individuals per division who self‐reported skin disease caused by specific allergenic foods. Ultimately, the survey included 970 respondents, with the in‐person data collection period spanning from December 2022 to July 2023.

### 2.4 Statistical analyses

To comprehensively analyze the collected data, we employed multiple statistical techniques. Initially, we used multiple response analysis to assess the frequency of the variety of potential food exposures linked to skin disease, allowing participants to identify multiple allergenic foods. Following this, we determined the final model using PCLR for binary outcomes. In the process of developing our model, we initially used PCA to discover uncorrelated latent variables, principal components (PCs), given the strong linkage between the original variables of different food items. Subsequently, after selecting PCs as independent variables and a binary outcome (Skin Disease by Allergenic Foods) as a dependent variable, we conducted PCLR. Notably, while we normally use PC regression for continuous outcomes, we can refer to it as PCLR for binary outcomes, which is free from multicollinearity. The PCLR model is as follows:

$$\text{Logit}\left(\text{Pr}\left(Y={1/\text{PC}}_{1}{,\text{PC}}_{2},\text{…}{,\text{PC}}_{k}\right)\right)=b_{0}+b_{1}{\text{PC}}_{1}+b_{2}{\text{PC}}_{2}+\text{…}+b_{k}{\text{PC}}_{k}$$

where *Y* is the binary outcome (Skin Disease by Allergenic Foods, “Yes = 1,” “No = 0”), and PCs are latent variables selected from PCA. PCs are a linear combination of original variables. Mathematically, it is like the following:

$$\begin{array}{lll} {\text{PC}}_{1}=a_{11} & X_{1}+a_{12} & X_{2}+\text{…}+\;a_{\text{1p}}X_{p}\\ {\text{PC}}_{2}=a_{21} & X_{1}+a_{22} & X_{2}+\text{…}+\;a_{2p}X_{p}\\ & \;‘ & \\ & \;‘ & \\ & \;‘ & \\ {\text{PC}}_{k}=a_{k1} & X_{1}+a_{k2} & X_{2}+\text{…}+\;a_{\text{kp}}X_{p} \end{array}$$

where *a*_*ij*_ represents the loadings and *X*_*s*_ denotes the original variables, which in our case are different food items.

To evaluate the robustness of our model, we employed additional statistical tests. Specifically, to assess the goodness of fit of the fitted model, we utilized the Hosmer and Lemeshow test. In this context, a *p* Value less than 0.05 would not be a better fitting model.^22^ Furthermore, to gauge the predictive capability of the model, we conducted receiver operating characteristic (ROC) curve analysis. Notably, greater area under the curve (AUC) value of the ROC curve shows higher predictive accuracy.^23^

We analyzed the data set using R version 4.3.2, a statistical tool.

## 3 RESULTS AND DISCUSSION

This study aimed to investigate the potential association between exposure to allergenic foods and the development of skin disease in Bangladesh. We conducted the survey across four major divisions—Dhaka, Sylhet, Rangpur, and Chattogram—to ensure a broad geographic representation with a response rate of 60%.

**Figure 1:**
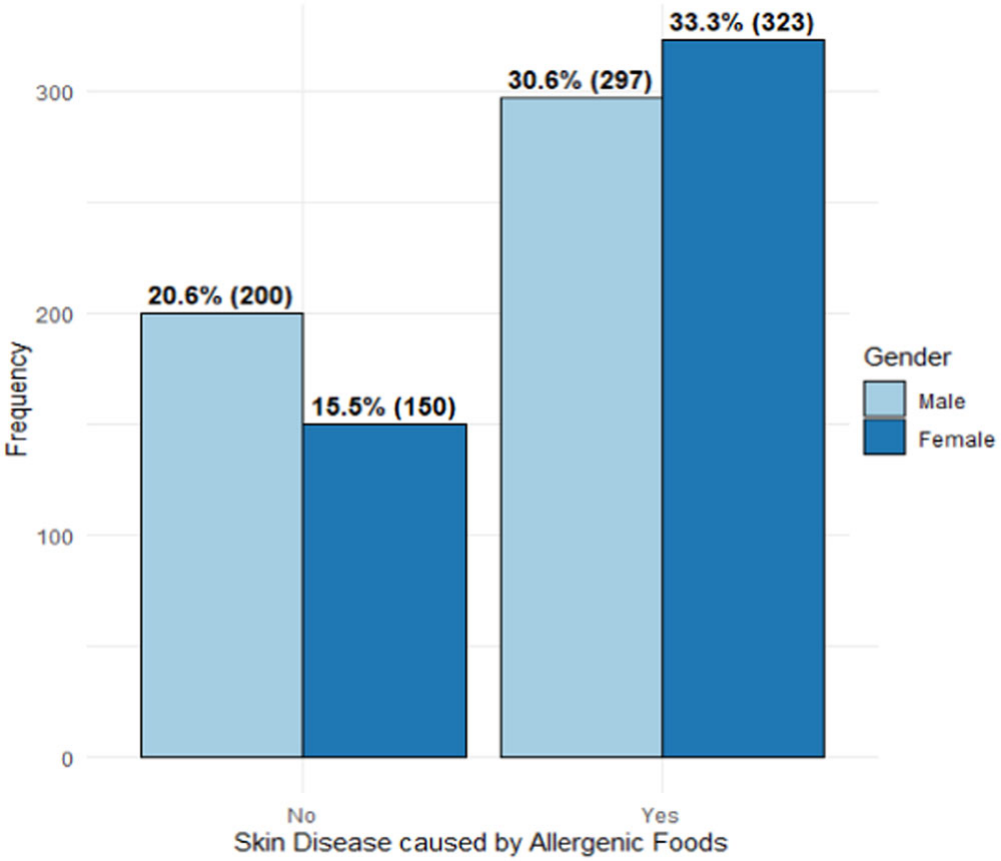
Presence of allergenic foods prompted skin disease by gender.

Figure 1 shows that 64% of respondents reported having skin disease during the survey period, which they believed was caused by certain allergenic foods. Of these individuals, 48% were male, and 52% were female. Conversely, among respondents who did not report any skin disease during the survey, males constituted a majority at 57%, while females represented 43% of the respondents.

**Figure 2:**
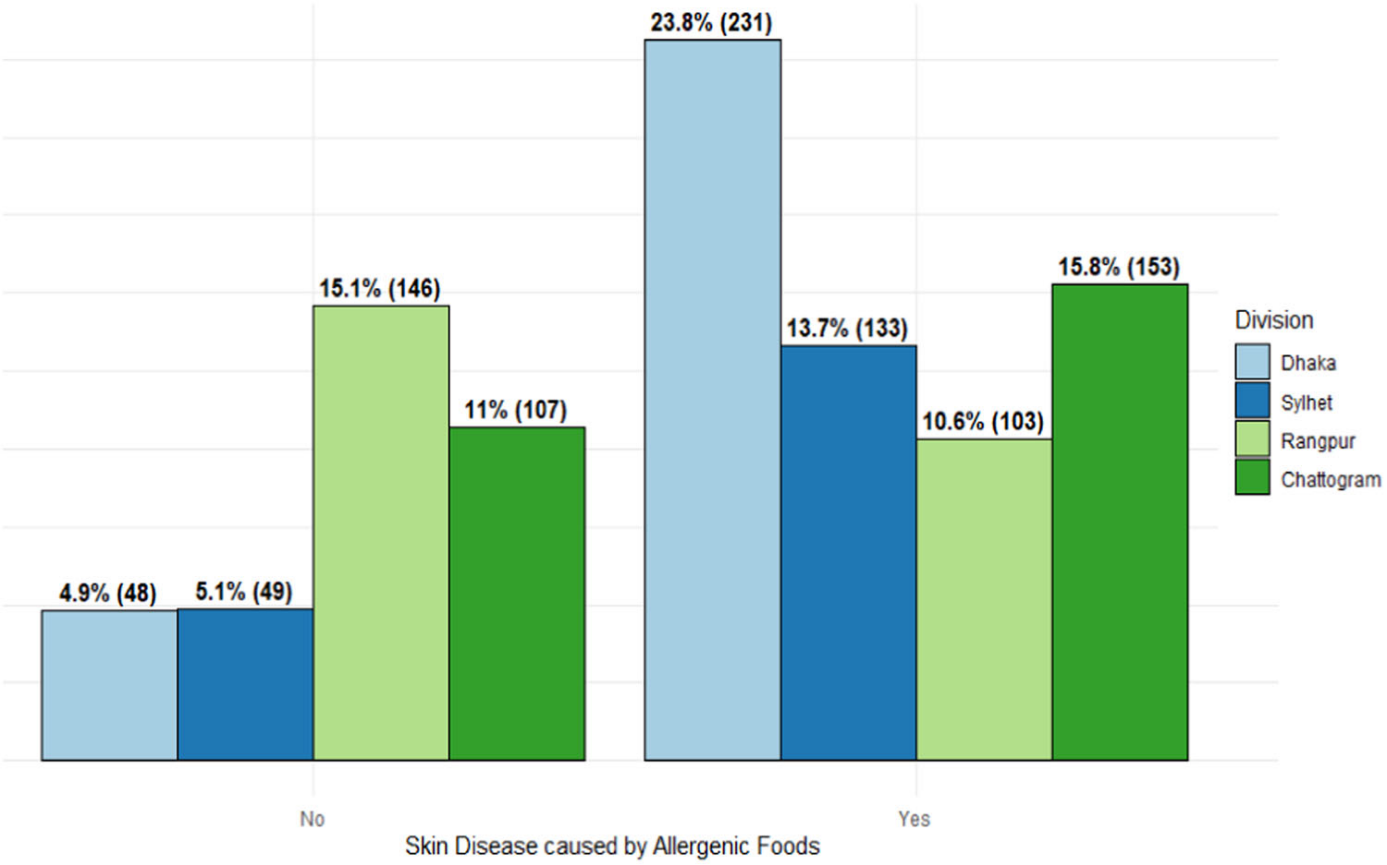
Presence of allergenic foods prompted skin disease by division.

Furthermore, Figure 2 depicts the distribution of respondents with the status of skin disease caused by allergenic foods, categorized by division. Notably, the majority of cases of respondents having skin disease during the survey were from Dhaka (37%), followed by Chattogram (25%), Sylhet (21%), and Rangpur (17%). Conversely, among respondents who did not report any skin disease during the survey, 42% of the cases were from Rangpur, followed by Chattogram (31%), Sylhet (14%), and Dhaka (14%).

We conducted a multiple response analysis, and Table 1 summarizes the results. Specifically, it shows the food items indicated by the respondents that might trigger their skin disease. Given that one respondent might refer to multiple responses for the occurrence of skin disease, the total valid responses increased from 452 to 1025. In the analysis, the “Responses” column uses the total number of responses (Total = 1025) as base value for the percentages, whereas the “Percent of cases” column uses the number of valid cases (452) as base while excluding the missing cases (518). Remarkably, Beef emerged as the most frequently cited allergen, with 67% of the respondents considering it as the allergenic food, accounting for 29.6% of all the answers. Following this, Brinjal (47.1%), Hilsa (45.8%), and Shrimp (23.7%) were also commonly reported, accounting for 20.8%, 20.2%, and 10.4% of all the answers, respectively. These findings resonate with previous research that identified brinjal, hilsa, and beef as the prevalent allergenic foods associated with skin disease in Bangladesh.^10^

**Table 1:** Frequencies of multiple responses of the food items.

Food items	Responses		Percent of cases
	*N*	%	
Hilsa	207	20.2%	45.8%
Shrimp	107	10.4%	23.7%
Brinjal	213	20.8%	47.1%
Spinach	18	1.8%	4.0%
Arum	22	2.1%	4.9%
Lentil	12	1.2%	2.7%
Corn	20	2.0%	4.4%
Beef	303	29.6%	67.0%
Mutton	30	2.9%	6.6%
Duck	19	1.9%	4.2%
Pineapple	15	1.5%	3.3%
Chicken's egg	16	1.6%	3.5%
Duck's egg	43	4.2%	9.5%
Total	1025	100.0%	226.8%

Abbreviation: *N*, frequency.

To further investigate the underlying factors related to allergenic foods causing skin disease, we performed PCA, as shown in Table 2. This table illustrates the correlations between the original data and each PC. In our analysis, we focused on the most significant relationships by selecting the correlation of the original variables (food items) with a threshold of |0.30| or greater, suppressing values smaller than this threshold. As a result of this approach, six PCs were identified, collectively accounting for 62.6% of data variation. Specifically, PC_1_, PC_2_, PC_4_, PC_3_, PC_5_, and PC_6_ individually accounted for 14.9%, 10.7%, 10.2%, 9.7%, 9.2%, and 8% of data variation, respectively. Notably, compared to continuous variables, categorical variables exhibit more pronounced variation.^24^ Given that PCs are continuous variables, this variability is reflected in Table 2. According to our findings, the first PC (PC_1_) accounted for the highest variation in the data. Consequently, PC_1_ emerged as the most vital among the six PCs identified. Upon closer examination, we observed that PC_1_ correlates with three original factors. Specifically, the value of PC_1_ rises with Brinjal, Beef, and Hilsa, respectively. This correlation suggests that these three criteria exhibit notable variation. Furthermore, PC_1_ largely reflects Hilsa, as evidenced by its substantial correlation value of 0.791. Hence, respondents with skin disease are more likely to consume Hilsa.

**Table 2:** Loadings (suppressed <|0.30 | ) of principal component analysis.

Food items	Principal components					
	PC_1_	PC_2_	PC_4_	PC_3_	PC_5_	PC_6_
Hilsa	0.791					
Shrimp			0.434	0.501		
Brinjal	0.781					
Spinach				0.752		
Arum			0.616			
Lentil				0.545		
Corn						0.944
Beef	0.787					
Mutton					0.753	
Duck		0.800				
Pineapple			0.711			
Chicken's egg					0.753	
Duck's egg		0.776				
Proportion variance	0.149	0.107	0.102	0.097	0.092	0.080
Cumulative variance	0.149	0.256	0.357	0.454	0.546	0.626

We further employed PCLR to explore the contribution of underlying patterns to the development of skin disease. PCA was used to reduce the dimensionality of the data by transforming the original variables into PCs. The six PCs were then used in logistic regression model to evaluate their association with the development of skin disease. We ran a multiple logistic regression model, considering responses to Skin Disease by Allergenic Foods (Yes = 1, No = 0) and scores of six PCs as covariates. Initially, we ran a saturated model and found that PC_4_ was the most insignificant. Additionally, the saturated model did not show better fit of the data as the *p* Value of the model from the Hosmer−Lemeshow (HL) test was computed as 0.042. After deleting the PC_4_, we ran the model again, referring to it as the reduced model, and found an increment of about 0.018 of the *p* Value for the HL test, or 0.06, indicating that the reduced model fits the data well.

**Figure 3:**
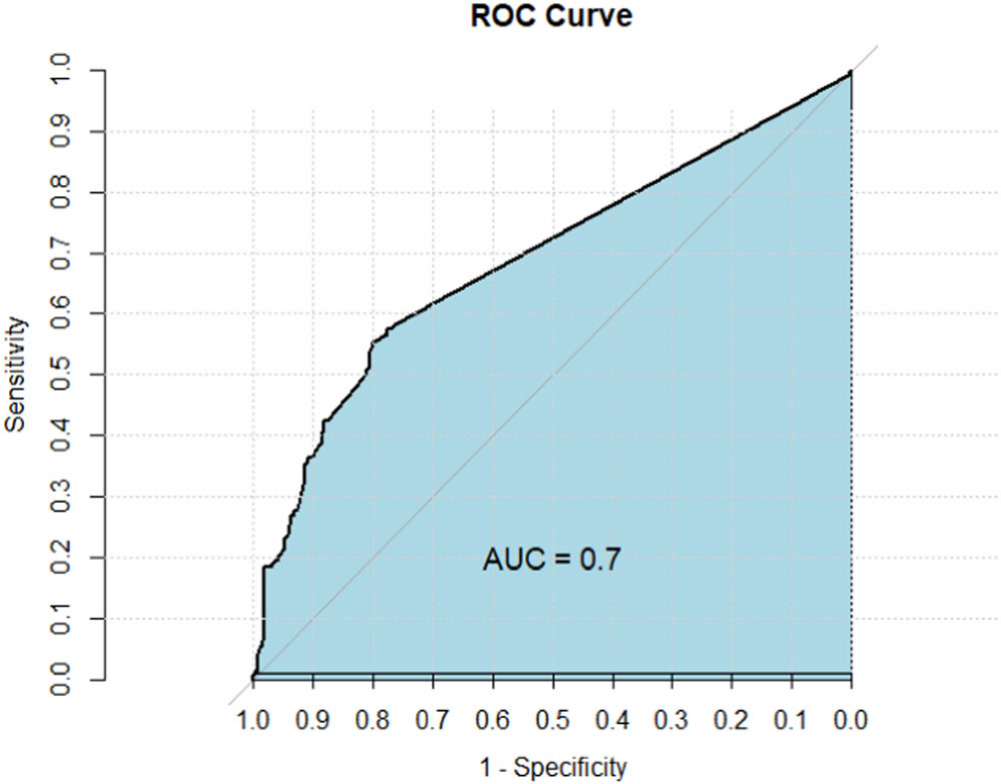
ROC curve for model selection.

Figure 3 represents the receiver operating characteristic (ROC) curve that displays the area under the curve (AUC) value. The ROC curve had an AUC value of 0.7, indicating moderate predictive accuracy and reflecting the significant impact of the explanatory variables on the reduced model's prediction.

Table 3 presents odds ratios and 95% confidence intervals (CI) of the PCs from the reduced model. Notably, it shows that PC_1_ and PC_6_ were highly significant. Specifically, we found the OR of PC_1_ to be 2.256 (95% CI: 1.886, 2.700). This indicates that a one‐unit increase in the PC_1_ representing Hilsa, Beef, and Brinjal is associated with a 125.6% increase in the odds of having a skin disease caused by allergenic foods. Similarly, we found the OR of PC_6_ to be 1.342 (95% CI: 1.039, 1.489). In this case, it indicates that a one‐unit increase in the PC_6_ representing Corn is associated with a 34.2% increase in the odds of having a skin disease caused by allergenic foods. Our findings are consistent with previous research. For instance, foods that cause allergic reactions have been found to include hilsa, beef, and brinjal, among others.^10^ Additionally, beef and fish have been identified as allergenic foods in another study.^18^ Furthermore, various types of fish have been recognized as potential allergens in various studies.^15^, ^16^, ^19^ Interestingly, from a veterinary perspective, beef and corn have also been identified as allergenic foods, further corroborating our findings.^17^

**Table 3:** Principal component logistic regression.

Principal Components	B	SE	Wald	*p* Value	OR	95% CI for OR	
						Lower	Upper
PC_1_	0.814	0.092	79.138	0.000	2.256	1.886	2.700
PC_2_	0.152	0.084	3.328	0.068	1.165	0.989	1.372
PC_3_	0.178	0.092	3.748	0.053	1.194	0.100	1.430
PC_5_	0.061	0.079	0.597	0.440	1.063	0.910	1.242
PC_6_	0.294	0.115	6.504	0.011	1.342	1.039	1.489
Constant	0.710	0.077	84.987	0.000	2.033	1.071	1.683
Hosmer and Lemeshow Test				Chi‐square = 9.164			
				*df* = 4, *p* = 0.060			

Abbreviations: B, regression coefficient; CI, confidence interval; OR, odds ratio; SE, standard error.

### 3.1 Strengths of the study

1. The study used PCLR to analyze the data, which is a robust statistical technique that addresses multicollinearity issues commonly encountered in traditional logistic regression models.
2. The study used multiple response analysis to allow respondents to identify multiple allergenic foods, which is useful in capturing the complexity of dietary exposures and their potential cumulative effects on skin disease.
3. This study covers diverse geographic regions within a country.

### 3.2 Limitations of the study

1. The cross‐sectional nature of this study lacks longitudinal nature which limits the ability to infer causality between allergenic food exposure and skin disease.
2. Although the study covered four major divisions in Bangladesh, the findings might not be representative of other regions with different dietary habits and environmental conditions.
3. This research generalizes skin diseases without distinguishing between different types.
4. The reliance on self‐reported data might introduce biases, such as misreporting or underreporting of dietary intake and skin disease symptoms.

## 4 CONCLUSION

This study investigated the potential association between exposure to allergenic foods and skin disease development in Bangladesh. We uncovered several significant findings using a cross‐sectional survey approach and advanced statistical techniques, such as PCLR. The multiple response analysis revealed that Beef, Brinjal, Hilsa, and Shrimp were the most frequently reported allergenic foods associated with skin disease among the respondents.

The reduced model identified two highly significant PCs, PC_1_, and PC_6_, which correlated strongly with specific food items. PC_1_, representing a combination of Hilsa, Beef, and Brinjal, exhibited an odds ratio of 2.256, suggesting that individuals consuming these foods were 125.6% more likely to develop skin disease compared to nonconsumers. Similarly, PC_6_, primarily reflecting Corn, had an odds ratio of 1.342, indicating a 34.2% higher probability of skin disease development relative to non‐consumers of Corn.

Although these findings enhance our comprehension of the possible association between allergenic food exposure and skin health outcomes in Bangladesh, we must acknowledge certain limitations. The cross‐sectional nature of the study precludes causal inferences, and the reliance on self‐reported data may introduce biases. Additionally, the study's focus on specific geographic regions within Bangladesh may limit the generalizability of the results. Future research efforts should consider longitudinal study designs and incorporate clinical assessments to validate self‐reported data, thus enhancing the robustness of the findings. Moreover, expanding the geographic scope and investigating specific types of skin diseases could provide further insights into the nuanced relationships between allergenic foods and different dermatological conditions.

Despite these limitations, the present study underscores the importance of raising awareness about the potential impact of dietary choices on skin health in Bangladesh. By identifying common allergenic foods, this research lays the foundation for developing targeted interventions, educational campaigns, and dietary recommendations to improve the management and prevention of skin disease attributed to allergenic foods within the country.

## AUTHOR CONTRIBUTIONS

**Md. Abrar Ashfaq Khan**: Data curation; writing—original draft; investigation; visualization; writing—review and editing; software. **Md Rashed Babu**: Investigation; data curation; project administration. **Sumaiya Tasnim**: Data curation. **Atiya Tarannum**: Data curation. **Mohammad Anamul Haque**: Investigation; validation; project administration. **Nahid Sultana**: Investigation; validation; project administration. **Mohammad Ohid Ullah**: Conceptualization; methodology; formal analysis; supervision; funding acquisition; investigation; writing—review and editing; validation; project administration; resources. All authors have read and approved the final version of the manuscript.

## CONFLICT OF INTEREST STATEMENT

The authors declare no conflict of interest.

## ETHICS STATEMENT

This study was approved by the SUST Research Center of Shahjalal University of Science and Technology, under Project ID: PS/2022/2/34, titled “Identifying the factors related to skin diseases in Bangladesh: A cross‐sectional study.” We conducted the study over the period 2022−2024. Further verbal informed consent was obtained from the respondents for their anonymized information to be published in this article. The questionnaire, which includes the informed consent, is provided in the Appendix A.

## TRANSPARENCY STATEMENT

The lead author Md. Abrar Ashfaq Khan affirms that this manuscript is an honest, accurate, and transparent account of the study being reported; that no important aspects of the study have been omitted; and that any discrepancies from the study as planned (and, if relevant, registered) have been explained.

## Data Availability

The Corresponding Author Mohammad Ohid Ullah had full access to all of the data in this study and takes complete responsibility for the integrity of the data and the accuracy of the data analysis.

## APPENDIX A

Questionnaire with Informed Consent

Survey on Skin Disease by Allergenic Foods

The questionnaire is developed for apart of the project titled as “Identifying the Factors Related to Skin Diseases in Bangladesh: A Cross‐sectional Study.” This project is funded by SUST Research Center, SUST, Sylhet, Bangladesh. **The information provided by you in this questionnaire will be used for research purposes only. It will not be used in a manner which would allow identification of your individual responses**.

**Table:**

1.		ID		
2.		Gender	Female	□
			Male	□
3.		Division	Dhaka	□
			Sylhet	□
			Rangpur	□
			Chattogram	□
4.		Presence of Skin Disease during Survey	Yes	□
			No	□
If “**Yes**” in the 4^th^ information, proceed further!				
5.		Do you think that your skin disease is prompted by allergenic food(s)?	Yes	□
			No	□
If “**Yes**” in the 5^th^ information, proceed further!				
Please mention the allergenic food(s) below that is/are responsible for your skin disease!				
6.		Arum	Yes	□
			No	□
7.		Beef	Yes	□
			No	□
8.		Brinjal	Yes	□
			No	□
9.		Chicken's egg	Yes	□
			No	□
10.		Corn	Yes	□
			No	□
11.		Duck	Yes	□
			No	□
12.		Duck's egg	Yes	□
			No	□
14.		Hilsa	Yes	□
			No	□
16.	Mutton		Yes	□
			No	□
17.	Shrimp		Yes	□
			No	□

Please write the name of any other allergenic food(s) that is/are responsible for your skin disease.
